# Mechanistic understanding of the adsorption and thermodynamic aspects of cationic methylene blue dye onto cellulosic olive stones biomass from wastewater

**DOI:** 10.1038/s41598-020-72996-3

**Published:** 2020-09-28

**Authors:** Mohammad A. Al-Ghouti, Rana S. Al-Absi

**Affiliations:** Department of Biological and Environmental Sciences, College of Arts and Sciences, Qatar University, P.O. Box 2713, Doha, State of Qatar

**Keywords:** Environmental sciences, Environmental chemistry

## Abstract

In the current study, the mechanistic understanding of the adsorption isotherm and thermodynamic aspects of cationic methylene blue (MB) dye adsorption onto cellulosic olive stones biomass from wastewater were investigated. The batch adsorption of MB onto the olive stones (black and green olive stones) was tested at a variety of pH, dye concentrations, temperatures, and biomass particle sizes. The adsorption thermodynamics such as Gibbs free energy, enthalpy, and entropy changes were also calculated. Moreover, the desorption studies of MB from the spent olive stones were studied to explore the re-usability of the biomasses. The results revealed that under the optimum pH of 10, the maximum MB uptake was achieved i.e. 80.2% for the green olive stones and 70.9% for the black olive stones. The green olive stones were found to be more efficient in remediating higher MB concentrations from water than the black olive stones. The highest MB removal of the green olive stones was achieved at 600 ppm of MB, while the highest MB removal of the black olive stones was observed at 50 ppm of MB. Furthermore, for almost all the concentrations studied (50–1000 ppm), the MB adsorption was the highest at the temperature of 45 °C (*P* value < 0.05). It was shown by the Fourier transform infrared that the electrostatic interaction and hydrogen bonding were proposed as dominant adsorption mechanisms at basic and acidic pH, respectively. While the hydrophobic-hydrophobic interaction was a dominant mechanism at neutral pH. The thermodynamic studies revealed that the adsorption process was endothermic, spontaneous, and favorable. Moreover, the real wastewater experiment and the desorption studies showed that the green and black olive stones were a cost-effective and promising adsorbents for MB remediation from wastewater on account of their high adsorption and desorption removal capacities.

## Introduction

With the increase in global populations and developments, extensive pressure on industries is increasing. Specifically, demands on textile industries to meet the human needs for fabrics have been posing a challenge in many countries^1–3^. Textile industrial processes consume large quantities of water and produce huge amounts of wastewater that are packed with organic and inorganic chemicals such as dyes^4,5^. It has been recently estimated that the production of one kilogram of fabric requires the consumption of 95–400 L of water^6^. Dyes are generally soluble and are lost to effluents, which pose serious dangers to the environment^7–9^. A textile laundry wastewater, as an example, also constitutes a considerable amount of municipal sewage discharge and is among the major sources of hazardous dyes in the environment.

Methylene blue (MB), commonly known as basic blue 9, is a cationic soluble dye or stain that comes in the form of green/blue powder or crystalline solid which dissociates in aqueous solutions as chloride ions and cations. It is used in various fields such as medicine, biology, and chemistry^10^. However, MB is mostly used in textile industries for coloring fabrics. Common names of MB are methylthionine chloride, Aizen methylene blue, and Chromosmon. The IUPAC nomenclature is 3,7-bis(Dimethylamino)-phenothiazin-5-ium chloride. The molecular formula of MB is C_16_H_18_ClN_3_S and the relative molecular mass in the anhydrous form is 319.85^11^. Despite the beneficial uses of MB in science, the dye has harmful impacts on humans and the environment due to its water solubility. MB is toxic, a mutagenic agent, and suspected to be a carcinogenic dye^12^.

To overcome the impacts of dyed textile wastewater, various treatment methodologies have been recognized. Some of the common treatment technologies for textile wastewater are either chemical or physical treatments, which include floatation of pollutants on the surface of the water, coagulation/flocculation/sedimentation of pollutant particles, chemical oxidation, precipitation, solvent extraction, irradiation and membrane-based separation of contaminants from water^13–16^. The drawbacks of the conventional treatments outweigh their treatment abilities. For example, sludge formation, harmful emissions, heavy metal productions as well as high costs, malfunction risks and energy requirements make the employment of the traditional textile wastewater treatment methods challenging^14,17,18^.

A promising color remediation technique from wastewater to overcome the challenges associated with the conventional treatments is adsorption. The process of adsorption employs the adherence of molecules of liquid, gas, or dissolved solids (adsorbate) to a surface of a substance (adsorbent).

Adsorption can be achieved by using a variety of adsorbents. Adsorption typically involves covalent bonding between the adsorbate and adsorbent, which could be reversed by certain conditions. The low cost, simple operation, flexibility, technical feasibility, and typical low waste generation are common characteristics of adsorption processes^19^. Conventionally, adsorption on activated carbon has been excessively used to treat dyes from wastewater. Despite the popularity of activated carbon in adsorption, it has poor regeneration capacities, it can toxic to many living organisms, and its use and re-use are costly^7,20,21^. Therefore, to ensure sustainability and eco-friendly methods of adsorption, alternatives to activated carbon are studied. Agricultural wastes and cellulosic biomass provide a great potential for wastewater remediation as substitutes for activated carbon in the adsorption technique^14,19^. Bio-composites prepared from agricultural wastes such as mango stones^22^, and peanut wastes^23^, have been proved to be efficient adsorbents for crystal violet dyes from aqueous solutions. Similarly, rice bran composites have also been prepared and successfully utilized for adsorption and removal of malachite green dye^24^. Date seeds, wheat bran, dolomite, fruit peels are also some of the examples of bio-products that have been utilized as adsorbents^8,25–27^.

Environmentally safe, efficient, and cost-effective adsorbents are olive stones and cellulosic biomasses. In 2010, olive oil production was around 2,881,500 metric tons globally, and between 1990 and 2010, a 78% increase in the worldwide olive oil consumption was estimated^26^. Moreover, during 2017 and 2018, it was estimated that the global consumption of olives reached 3.008 × 10^6^ tons^28^. Generally, olives come in many shapes, sizes, and colors depending on the cultivation region conditions. However, olives share the same structure: the skin (epicarp), pulp (mesocarp), and the stone (woody endocarp) which holds the seed. The composition of the pulp is similar to all types of olives, but the ratios of nutrients are different. There are oils, fats, and sodium in black olives, which results in higher amounts of carbon content than green olive stones. Olive stones are lignocellulosic biomasses with a main composition of hemicelluloses, celluloses, lignin, fats, phenols, polysaccharides. As a result of the massive production of olives, large quantities of olive stones are produced as waste^28,29^. The growing knowledge about the high-value composition of olive stones and the interests of many countries in keeping the environment as clean as possible has led to many innovative uses of olive stones. Some of these applications include combustion, extraction of bio-oils, and skin exfoliate material. An interesting expanding application of olive stones is employing them as heavy metals, and dyes adsorbents in wastewater treatment plants^25,29,30^.

In this paper, the mechanistic understanding of the adsorption and thermodynamic aspects of cationic MB dye onto cellulosic olive stones biomass from wastewater were investigated. The adsorption of MB dye from water by olive stones obtained from unmodified raw granular black and green olive stones was studied. This is due to the mentioned drawbacks of using activated carbon, the high cost as well as pollution risks of chemically treating adsorbents. Thus, the untreated olive stones represent a cheaper, environmentally friendly, and economically stable method to remove MB from wastewater. This could be effectively done to sustainable treat wastewaters from MB, expand the applications of olive stones, and maximize their use.

Therefore, the objectives of the study are to (i) study the physical and chemical characterizations of two samples of olive stones obtained from black and green olives using a scanning electron microscope (SEM), Fourier-transform infrared spectrophotometer (FTIR), carbon, hydrogen, and nitrogen (CHN), pH of solution (pH_solution_), as well as bulk and particle densities. This would give insights regarding the physical structure of the adsorbents and their chemical composition that enables them to adsorb MB dye, (ii) perform MB batch adsorption isotherm experiments onto olive stones as adsorbents, (iii) evaluate the effects of a variety of pH, dye initial concentrations, temperatures, and adsorbents particle size on the adsorption capacity of MB onto the olive stones. Finally, the application of the green and black olive stones as adsorbents to real wastewater was also examined.

## Materials and methods

### Adsorbents collection and preparation

Two representative samples of black and green olives belonging to the same plant species were collected randomly from different local markets. The olive stones were harnessed from the collected olive samples. Around 100 g of the green and black olive stones were collected. The collected olive stones were then washed thoroughly with distilled water to remove any impurities and then roasted at 130 °C for 24 h. After that, the samples were mechanically crushed and sieved using a sieve shaker with four mesh size ranges (2.50–1.00, 1.00–0.50, 0.50–0.250, and 0.250–0.125 mm). The sieved samples were then stored in 50 mL lidded glass bottles at 25 °C that were pre-washed with distilled water and dried to remove any impurities.

### Characterization of the adsorbents

The adsorbents were characterized using a scanning electron microscope (SEM) (JEOL, MP-08130T00L5), Fourier transform infrared spectroscopy (FTIR), as well as carbon, hydrogen, and nitrogen (CHN) (Flash 2000 Organic Elemental Analyzer). The pH of the original solution (pH_solution_) and bulk and particle densities were also carried out for the adsorbents. The ${\text{pH}}_{{{\text{solution}}}}$ was prepared by adding 0.03 g of the adsorbent in 30 mL distilled water, then shaken for 24 h at 150 rounds per minute (rpm) at 25 °C. The pH was measured prior to and after 24 h using a pH meter (Jenway, model 370 pH/Mv meter). The bulk density test is defined as the mass of particles divided by the total volume that they occupy. The test of bulk density was conducted by transferring 2.0 g of the adsorbent (particle size of 0.50–0.250 mm) to a 10.0 mL graduated cylinder and reading the initial volume of the sample at 25 °C. The cylinder was tapped gently to allow the adsorbent to settle. After the settlement of the sample, the second volume was obtained. Using Eq. (1), the bulk density of the adsorbents was calculated^31^. Similarly, the particle density of the adsorbents is defined as the mass of particles divided by the total volume of water that they displaced. The particle density of the adsorbents was obtained by adding 4.0 mL of distilled water into a 10.0 mL graduated cylinder and then transferring 2.0 g of the same sample at 25 °C. After the settlement of the sample, a second reading of the volume was obtained. The calculation of the particle density of the adsorbents was done using Eq. (2)^31^.

1 $${\text{Bulk}}\;{\text{density}}\,\left( {{\text{g/cm}}^{3} } \right) = \frac{{{\text{mass}}\;{\text{of}}\;{\text{adsorbent}}}}{{{\text{final}}\;{\text{volume}}\;{\text{of}}\;{\text{adsorbent }}}}$$

2 $${\text{Particle}}\;{\text{density}}\,\left( {{\text{g/cm}}^{3} } \right) = \frac{{{\text{mass}}\;{\text{of}}\;{\text{adsorbent }}}}{{{\text{final}}\;{\text{volume}}\;{\text{of}}\;{\text{water}}\;{\text{displaced}}}}$$

### MB stock solution preparation

An MB stock solution of 1000 ppm was prepared by adding 1 g of MB in a 1 L volumetric flask, then filling it with distilled water till the mark. The MB (C_16_H_18_N_3_SCl, molecular weight: 319.85 g/mol) standard powder was obtained from Fluka Chemia (number, 66721) and was packed in Switzerland. Distilled water was added slowly to the volumetric flask while shaking it gently to allow the MB to completely solubilize. A linear calibration curve of MB was conducted by preparing MB concentrations of 0, 1, 2, 3, 4, and 5 ppm. The absorbance was measured under the maximum wavelength of 663 nm using a visible spectrophotometer (PerkinElmer Lamda 25 UV/VIS spectrophotometer). The absorbance of each concentration was recorded and a curve of MB concentration vs. absorbance was plotted.

### Adsorption isotherm batch experiments

Batch adsorption experiments were performed under different pH values (pH of 2, 4, 6, 8, and 10), temperatures (25, 35 and 45 °C), initial dye concentrations (50, 100, 200, 300, 400, 600, 800 and 1000 ppm) and particle sizes (1.00–0.50, 0.50–0.250 and 0.250–0.125 mm). The batch adsorption experiments were conducted in 100 mL lidded glass beakers, each with 50 mL MB and 0.05 g of olive stones (black and green olive stones). To ensure quality control and no experimental errors, two trials and blanking of each batch experiment were conducted. The batch experiments were conducted using an incubator shaker (Shaking Incubator, MODEL: SSI10R-2, Orbital-Shaking) under a constant speed of 150 rounds per minute (rpm) for 24 h (the time needed to reach equilibrium). The supernatants were then filtered and analyzed using a visible spectrophotometer (PerkinElmer Lamda 25 UV/VIS spectrophotometer) to determine the final dye concentration after the adsorption process. The spent adsorbents were also collected and allowed to dry at room temperature for further analysis using SEM, FTIR, surface area, as well as bulk and particle density techniques.

For the effect of pH, the particle size of 0.50–0.250 mm of the adsorbents and concentration of 1000 ppm MB were chosen. The different pH values were adjusted by adding sodium hydroxide (NaOH, 0.05 M) and hydrochloric acid (HCl, 0.05 M). For the effect of initial MB concentration and temperature, the particle size of 0.50–0.250 mm of the adsorbents was chosen. The pH value was adjusted to 10 as it was found to be the optimum pH value from the results of the previous experiment (effect of solution pH).

The effectiveness of the black and green olive stones as adsorbents was also evaluated for the treatment of real wastewater (pH 8.3). 600 mg/L of MB was chosen as a model concentration. The sample of wastewater was collected from a laundry center located in Doha, Qatar.

#### MB desorption study

The desorption studies were carried out by adding the spent black and green olive stones (MB-loaded; initial MB concentration of 600 mg/L) to 50 mL of acidic mixtures of acetic acid and ethanol (%vol) (10:1, 5:1, and 1:1). The mixture was then shaken at 25 °C and 150 rpm for 24 h. The supernatants were then filtered and analyzed using a visible spectrophotometer (PerkinElmer Lamda 25 UV/VIS spectrophotometer) to determine the final dye concentration.

### Analysis of the results

Studying the morphology and the chemical structure of the adsorbents after adsorption is necessary to understand the mechanisms of MB adsorption onto the adsorbents^32–34^. For these reasons, SEM, FTIR, and CHN as well as bulk and particle densities were examined for the olive stones involved in the adsorption process of MB. Moreover, the Langmuir, Freundlich, Dubinin–Radushkevich, and Temkin isotherm models were applied to understand the interactions between the adsorbate and adsorbent^35^. In addition, the behavior of the adsorption process in terms of favorability, reversibility, and energy was acknowledged using adsorption thermodynamics. The Gibbs free energy (∆*G*°), enthalpy (∆*H*°), and entropy (∆*S*°) were calculated to determine the adsorption process thermodynamics. Gibbs free energy is represented by Eq. (3).

3 $$\Delta G^{\circ } = - RTLnK_{L}$$

where R is the universal gas constant value of 8.314 J/mol.K, T is the absolute temperature in Kelvins and K_L_ is Langmuir isotherm constant, which can be expressed as standard enthalpy and entropy changes of adsorption as functions of temperature.

The relationship between temperature and K_L_ is shown in the following formula known as Van't Hoff equation Eq. (4):

4 $$LnK_{L} = - \frac{{\Delta {\text{H}}^{\circ } }}{RT} + \frac{{\Delta {\text{S}}^{\circ } }}{R}$$

where a plot of lnK_L_ vs. 1/*T* can be used to determine the values of ∆*H*° and ∆*S*° from the slope and intercept^32,35–37^.

Finally, single, and double factor two-way ANOVA was conducted using Microsoft excel for the results of the batch experiments. The ANOVA test determines whether there is any significant difference between the means of samples. In addition, this test allows for understanding the interactions between two independent variables on the dependent variable.

## Results and discussion

### Characterization of the adsorbents

#### Scanning electron microscope (SEM)

**Figure 1:**
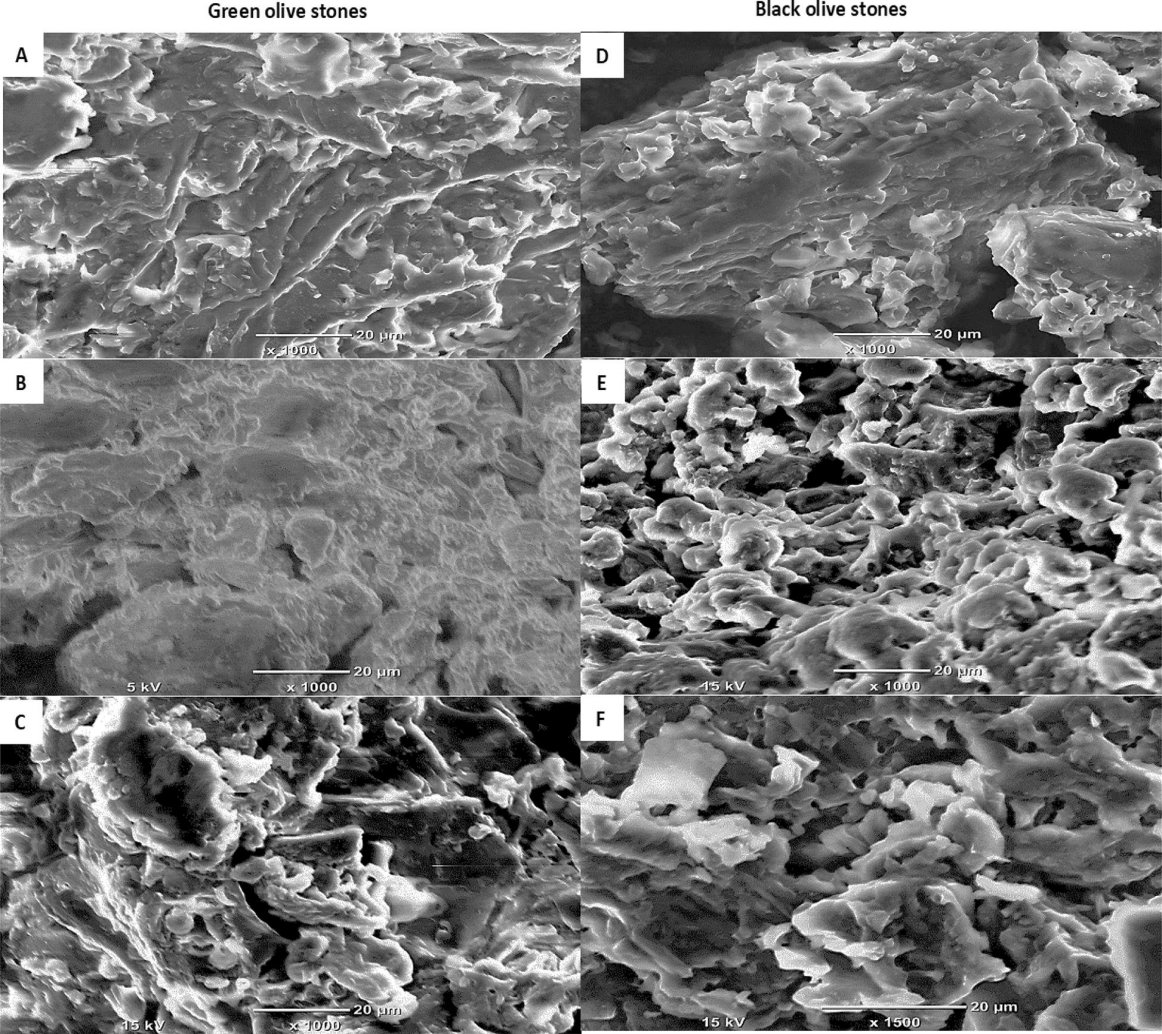
SEM images of the green and black olive stones of 0.50–0.250 mm size before and after MB adsorption. (**A**) Green olive stones at 25 °C. (**B**) Green olive stones after MB adsorption (1000 ppm MB). (**C**) Green olive stones after MB adsorption (600 ppm MB), (**D**) Black olive stones at 25 °C. (**E**) Black olive stones after MB adsorption (1000 ppm MB). (**F**) Black olive stones after MB adsorption (600 ppm MB).

Figure 1A–F shows the scanning electron microscopy (SEM) of the green and black olive stones before and after MB adsorption. The SEM images (Fig. 1A–F) give an overview of the surface morphology of olive stones as well as the changes that occur due to the MB adsorption^38^. The surface morphology of the green and black olive stones appears to have pores with different shapes and sizes. Also, the SEM reveals the presence of cavities on the green and black olive stones, which is a direct indication of the high surface area of the adsorbents. However, the black olive stones surface morphology appears to be smoother with less cracks and evident pores than the green olive stones. The same behavior was observed by Rizzi et al.^26^. After the adsorption of MB onto the olive stones, the pores of the green olive stones (Fig. 1B) appear to be blurry, indicating the filling of MB. On the other hand, the SEM images of the black olive stones (Fig. 1E) appear to be less blurry and clearer. This could be explained by less surface morphology changes or adsorption onto the black olive stones under the same conditions. Moreover, the surface morphology changes of the green and black olive stones before and after the MB adsorption of an initial MB concentration of 600 ppm (Fig. 1C,F) are clearly displayed. When comparing the SEM images of the stones involved in the adsorption of MB at an initial concentration of 600 ppm with the olive stones before MB adsorption, it is noticeable that the pores are still large in size, defined in structure and fairly clear. However, Fig. 1C,F shows that the black olive stones became less clear than the green olive stones after the adsorption of MB at an initial dye concentration of 600 ppm. This draws attention to the potentially significant role of the initial dye concentration in the adsorption of MB and changing the surface morphology of olive stones. In a study done by Udden et al.^39^ on the adsorption of MB onto mature mango leaves, results of SEM analysis were obtained. SEM images were taken for the mature mango leaves before and after adsorption and found obvious changes in the surface morphology of the mature mango leaves. The pores of the mature mango leaves appeared to be significantly blurry after the adsorption of MB. In another study conducted by Borhade and Kale^38^, calcined eggshells involved in the adsorption of three dyes (Rhodamine B, Eriochrome black T, and Murexide) were analyzed for SEM. The images of SEM revealed that after the adsorption of the dyes onto the calcined eggshells, the surface morphology of the adsorbent changed dramatically. The pores of the calcined eggshells were blurry and more packed together after the adsorption of the dyes.

#### Fourier-transform infrared (FTIR)

**Figure 2:**
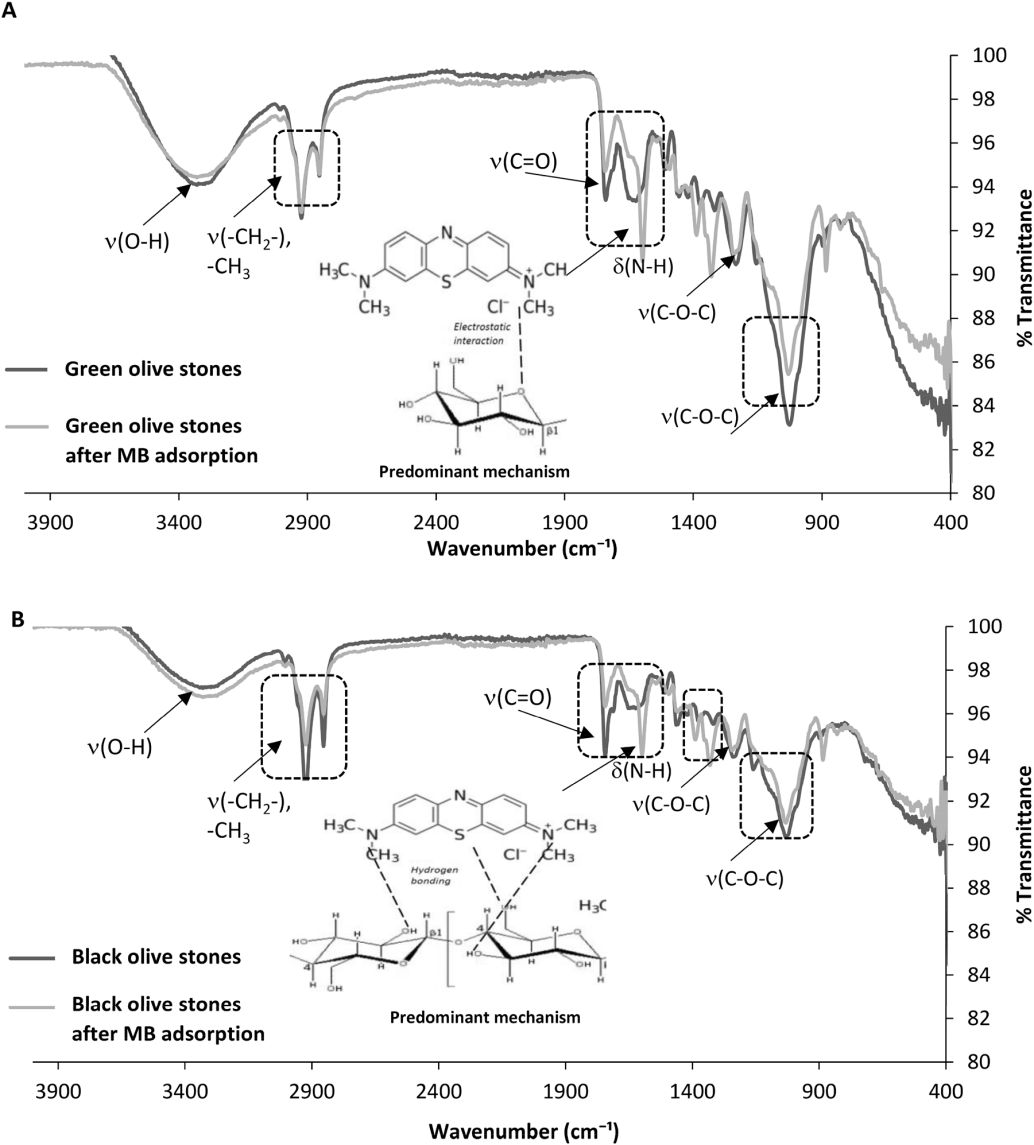
FTIR spectra of the green and black olive stones before and after MB adsorption. (**A**) Green olive stones and (**B**) black olive stones. Experimental conditions: concentration: 600 ppm, pH 10, temperature: 15 °C, volume: 50 mL, adsorbent mass: 0.05 g, particle size: 0.50–0.250 mm, and contact time: 24 h.

The determination of organic compounds, molecular structure, chemical bonding, and mainly functional groups of any organic material is carried out through FTIR. FTIR analysis gives insights regarding the specific functional groups in olive stones, which are involved in the remediation of MB. Figure 2A,B shows the FTIR spectra for the green and black olive stones before and after MB adsorption at an initial MB concentration of 600 ppm.

The peaks in the FTIR spectra for the green and black olive stones are indicated by boxes in Fig. 2. From the FTIR spectra of the green olive stones (Fig. 2A), it can be noticed that the absorbance pattern did not change significantly with the adsorption of MB. However, four peaks were obtained with the adsorption of MB onto the green olive stones. The peak at 2922 cm^−1^ is characteristic for C–H stretching and specifically for vibrations of –CH_3_ > CH_2_, CHO, and CH cellulose functional groups^40,41^. Proteins have characteristic FTIR peaks between 1800 and 1500 cm^−1^ where amide-I bands are between 1700 and 1600 cm^−1^ due to peptide bond stretching vibrations^42^. Moreover, N–H bending vibration peaks specific for amide-II occur in the region between 1600 and 1500 cm^−1^. The green olive stones at the study revealed peaks at 1692 and 1598 cm^−1^, which account for peptide bond stretching and N–H bending respectively due to MB adsorption^42^. Furthermore, the peak observed at 1033 cm^−1^ after the MB adsorption onto green olive stones (Fig. 2A) is mainly due to stretching vibrations caused by polysaccharides, specifically hemicelluloses. According to studies, the FTIR region between 1200 and 900 cm^−1^ is dominated by polysaccharides functional groups such as C–C, C–O, C–O–C, and C–O–P vibrations^40,41,43^.

On the contrary, the black olive stones showed more FTIR peaks (Fig. 2B) than the green olive stones after the adsorption of MB. This could be attributed to the presence of more functional groups responsible for the adsorption of MB for the black olive stones than the green olive stones^35^. This result is confirmed by the SEM images obtained and discussed for the olive stones in the previous section. Peaks were also obtained at 2922 and 2854 cm^−1^ for the black olive stones. This indicates the role of –CH_3_ > CH_2_, CHO, and C-H functional groups in the remediation of MB by the black olive stones^40^. Moreover, the peak for the black olive stones at 1742 cm^−1^ is characterized for the C=O functional group^36,39^, and at 1599 cm^−1^ is characterized for N–H bending. Figure 2B also shows a peak at 1330 cm^−1^ which corresponds to C-H asymmetric and symmetric bending^41,43^. The peak at 1031 cm^−1^ in Fig. 2B for the adsorption of MB is due to C–C, C–O, C–O–C, and C–O–P vibrations. For the green and black olive stones (Fig. 2A,B), the lack of peaks between 500–600 cm^−1^ indicates that the roles of alkyl halides functional groups such as C–Cl, C–Br, and C–I in the adsorption process of MB onto the adsorbents were not significant^40,41,43,44^.

#### Carbon, hydrogen, and nitrogen elemental analysis (CHN), pH_solution_, and Bulk and particle density

It is vital to establish a complete understanding of the composition of adsorbents, in particular, the carbon, hydrogen, and nitrogen contents. As mentioned earlier, carbonaceous materials allow efficient levels of adsorption to occur due to their porous nature and structure^21,45^. Table 1 shows the carbon, hydrogen, and nitrogen elemental analysis (CHN), pore size distribution, pH_solution_, along with the bulk and particle densities of the adsorbents. From Table 1, the black olive stones exhibit slightly more carbon, hydrogen, and nitrogen percentages than the green olive stones. The pH values for the green and black olive stones before and after MB adsorption were measured. It was concluded that the olive stones have a slightly acidic nature. However, the green olive stones exhibited a lower pH value than the black olive stones. Moreover, the pH value of the green olive stones decreased by 0.19 when the solution was homogenized under the influence of the mechanical shaker, while for the black olive stones, the pH value increased by 0.03.

**Table 1:** CHN elemental analysis, pH_solution_, pore size and surface area characterization, bulk density, and particle density for the olive stones.

Adsorbent	CHN elemental analysis		
	C%	H%	N%
Green olive stones	47.56	6.19	0.39
Black olive stones	48.12	6.37	0.55

	pH_solution_ (Experimental conditions: mass of adsorbent: 0.03 g, solution volume: 30 mL distilled water, temperature: 25 °C, and contact time: 24 h)		
	Before adsorption	After adsorption	∆pH
Green olive stones	5.82	5.62	0.19
Black olive stones	6.31	6.34	− 0.03

	Bulk density (g/cm^3^) (Experimental conditions: mass of adsorbent: 2 g, particle size: 0.5–0.250 mm, volume: 10 mL, and temperature: 25 °C)	Particle density (g/cm^3^) (Experimental conditions: mass of adsorbent: 2 g, particle size: 0.50–0.250 mm, volume: 4 mL, and temperature: 25 °C)	
Green olive stones	2	3	
Black olive stones	2.2	3.2	

	Pore size and surface area characterization		
	Volume of the macropores (cm^3^/g)	Volume of the mesopores (cm^3^/g)	Specific surface area (S_BET_) (m^2^/g)
Green olive stones	0.189 ± 0.009	0.0430 ± 0.0008	1.21 ± 0.04
Black olive stones	0.171 ± 0.009	0.0355 ± 0.0011	1.03 ± 0.06

The bulk density of any particulate material refers to the mass of the material divided by the volume it occupies, whereas the particle density is defined as the mass of the material divided by the volume of displaced water^31^. Furthermore, knowledge of spatial arrangement and packing of the green and black olive stones as well as their bulk densities will help in selecting the right adsorbent density for maximum cleanup of MB. The results showed that the black olive stones have slightly higher bulk and particle densities than the green olive stones. It was shown from the Table 1 that the surface area and the volume of pores of the green olive stones are quite higher than the black olive stones.

### Adsorption isotherms

#### Effect of the solution pH on the MB adsorption onto olive stones

**Figure 3:**
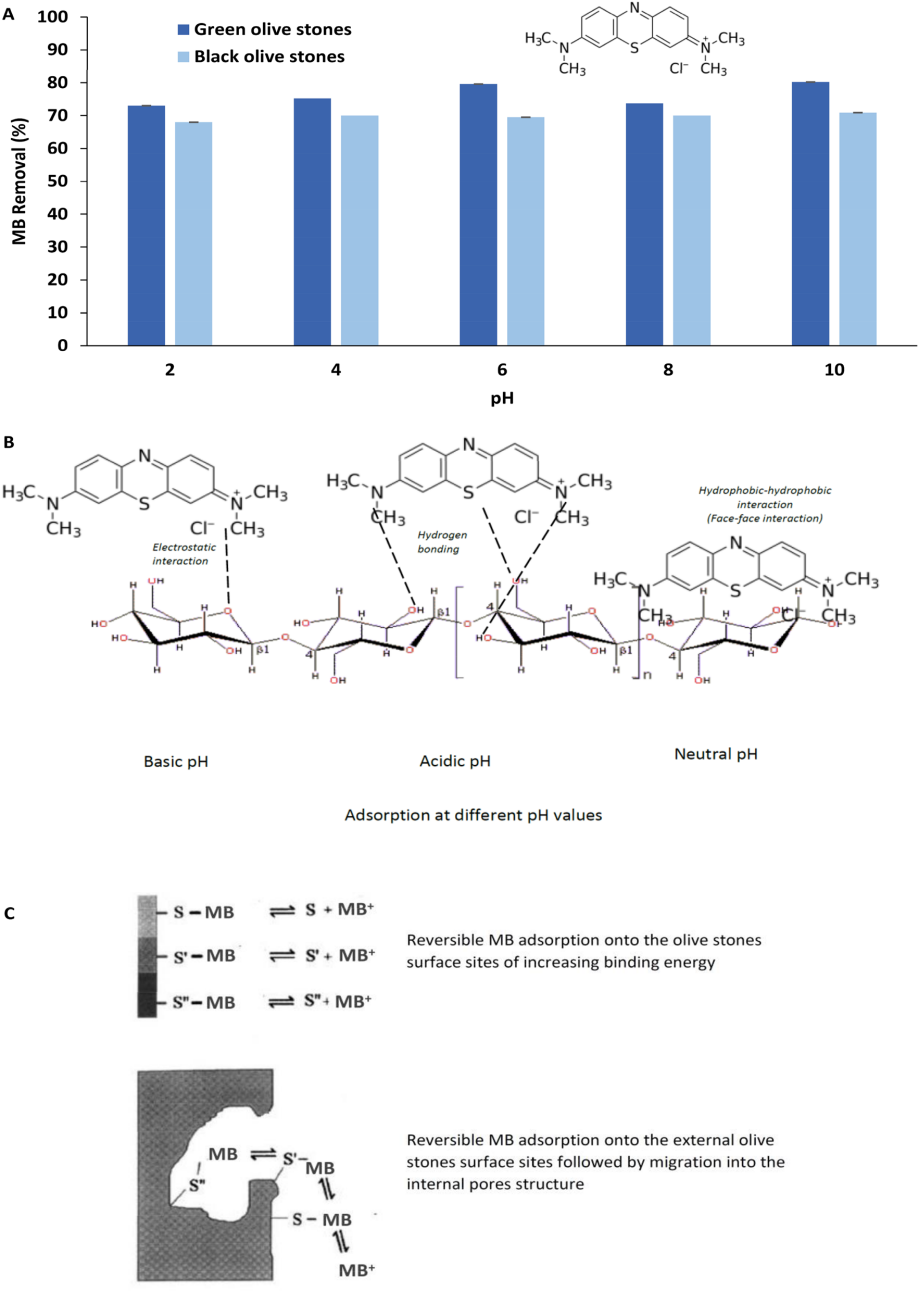
(**A**) Percentage removal of MB from water by the green and black olive stones at different pH values, (**B**) possible MB interactions onto the olive stones surface at different pH values, and (**C**) adsorption mechanisms for MB uptake by non-porous and porous solids (modified from^50^). Experimental conditions: particle size: 0.50–0.250 mm, adsorbent mass: 0.05 g, temperature: 25 °C, initial dye concentration: 1000 ppm, solution volume: 50 mL and contact time: 24 h.

Dye adsorption is largely affected by the solution pH. When dyes are present in water, they disassociate and ionize to form electrostatic charges in the solution^46^. The amounts and kinds of electrostatic charges that dyes will release in water are controlled by the pH of the solution^33^. Therefore, in an adsorption system, the extent to which a certain dye will be attracted and adhere to the adsorbent will vary with the pH. This is due to the fact that opposite electrostatic charges attract, while equal electrostatic charges repel. In an environmental adsorption clean-up method, it is important to study the effect of the solution pH on the extent of removal of the pollutant by the adsorbent^32,47^. For these reasons, the effect of the solution pH was the first parameter tested for the adsorption of MB onto the green and black olive stones. Figure 3 shows the percentage removal of MB by the olive stones at different pH values. The pH values examined for the adsorption of MB onto olive stones were 2, 4, 6, 8, and 10. The adsorption of MB onto the green and black olive stones was highest at pH 10 and lowest at pH 2 (*P* value ≥ 0.05). This means that when the solution is at the highest basic level, the highest percentage removal of MB can be achieved (80.2% for the green olive stones and 70.9% for the black olive stones). This can be attributed to the dominance of negative charges on the adsorbent surface, which leads to higher electrostatic attractions and adherence of MB due to its cationic nature^26,33^. Lower percentage removals obtained at acidic conditions (pH 2) can be explained by the fact that the adsorbent surface appeared positively charged for the cationic adsorbate, which lead to electrostatic repulsion and as a result, less adsorption to occur^26^. Similar results for MB and Alizarin Red S adsorption onto olive stones were obtained by a study conducted by Albadarin and Mangwandi^48^. It was found that the adsorption capacity of anionic Alizarin Red S was highest at pH of 3.28 while the maximum adsorption capacity of cationic MB was obtained at basic pH values. Furthermore, the adsorption of various dyes, including MB and malachite green onto activated charcoal was studied by Iqbal and Ashiq^49^. The study revealed that optimum adsorption of the anionic dyes occurred at low pH values, whereas for MB and malachite green (cationic dyes), the adsorption was highest at increased pH values. The study presents a further understanding of the effect of pH on the adsorption of pollutants according to their ionic nature.

Figure 3 also reveals that for the green olive stones, the percentage removal of MB was constantly enhanced as the pH increased (pH 2–6) then decreased at pH 8 (73.7%) followed by a sharper increase at pH 10. On the other hand, the percentage removal of MB for the black olive stones increased slightly from 68 to 70% when the pH changed from 2 to 4, then decreased very slightly to 69.5% at pH 6 followed by an increase at pH 8 and 10. Furthermore, it is worth mentioning that for the black olive stones, the same percentage removal was obtained at pH 4 and 8 (70%). From the overall trends of the percentage removal of MB for both types of olive stones, it is clear that the pH and ionic strength play a much greater effect on the adsorption of MB onto green olive stones when compared to the black olive stones. This can be observed from the larger changes in the percentage removals as the pH varied for the green olive stones than the black olive stones. It is worth mentioning that despite the less percentage removals of MB obtained at acidic pH values, the adsorption can still be considered highly efficient due to the overall high percentages achieved (all equal to or above 68%). Similar overall high adsorption percentages of disperse dye removal from aqueous solution by olive stones were obtained from a study done by Rizzi and others in 2017^26^. The study points attention towards the presence of various interaction forces of equal importance to the electrostatic interactions involved in the adsorption process. The suggested most popular interaction forces between adsorbents and adsorbate molecules are hydrophobic and hydrophilic forces, H-bonding, and van der Waals forces.

In order to study the effect of the solution pH on the MB adsorption onto the two types of olive stones in detail, two more pH related experiments were conducted. The first is measuring the natural pH of MB present in 50 mL of distilled water, which was found to be around 5. The second experiment involved a second measurement of the solution pH after the adsorption batch experiment was done. This allows a comparison of the pH before and after adsorption, which is important for drawing more reliable conclusions about the impacts of the pH on the adsorption mechanisms of olive stones^47^. The pH value adjusted prior to the MB adsorption by the olive stones is referred to in this study as initial pH and the pH value measured after the adsorption of MB by the olive stones is final pH. The difference between the two pH values was calculated as ∆pH.

It can be observed that for both types of olive stones there was a continuous increase in ∆pH as the initial pH increased from 2 to 10. In studying the effect of solution pH on the adsorption of MB onto olive stones, it was concluded that the pH value accounting for the highest percentage removal of MB onto olive stones was 10. It was also noted that the adsorption of MB onto olive stones generally favored basic pH values. As a result of the higher removal of MB onto olive stones at higher pH values, the final pH value of the system is observed to be somewhere in between the initial pH and the natural pH value of MB, which was found to be 4. This means that the surface of the olive stones moves toward reaching an equilibrium status after the adsorption towards the original pH of MB. For instance, the final pH values of the green and black olive stones involved in the adsorption of MB at an initial pH of 10 are 7 and 7.1 respectively. On the other hand, due to possibly less adsorption of MB onto the green and olive stones at acidic pH, the final pH values did not change significantly from the initial pH values. For example, at pH 2 (where the least percentage removals of MB were achieved for both types of olive stones) the final pH values were 2.2 and 2.4 for the green and black olive stones, respectively. The electrostatic interaction and hydrogen bonding were proposed as dominant adsorption mechanisms at basic and acidic pH, respectively. While the hydrophobic-hydrophobic interaction was a dominant mechanism at neutral pH. Figure 3B shows the possible MB interactions onto the olive stones surface at different pH values. Figure 3C illustrates the adsorption mechanisms for uptake of MB by non-porous and porous solids. It is assumed that the adsorption mechanism of the MB molecules could be achieved as a single-stage in which a rapid equilibrium on the surface of the adsorbent attains. The MB migration into the pores and bind with the higher-energy binding sites could also be attained as a slower stage^50^.

#### Effect of the initial MB concentration on its adsorption onto olive stones

**Figure 4:**
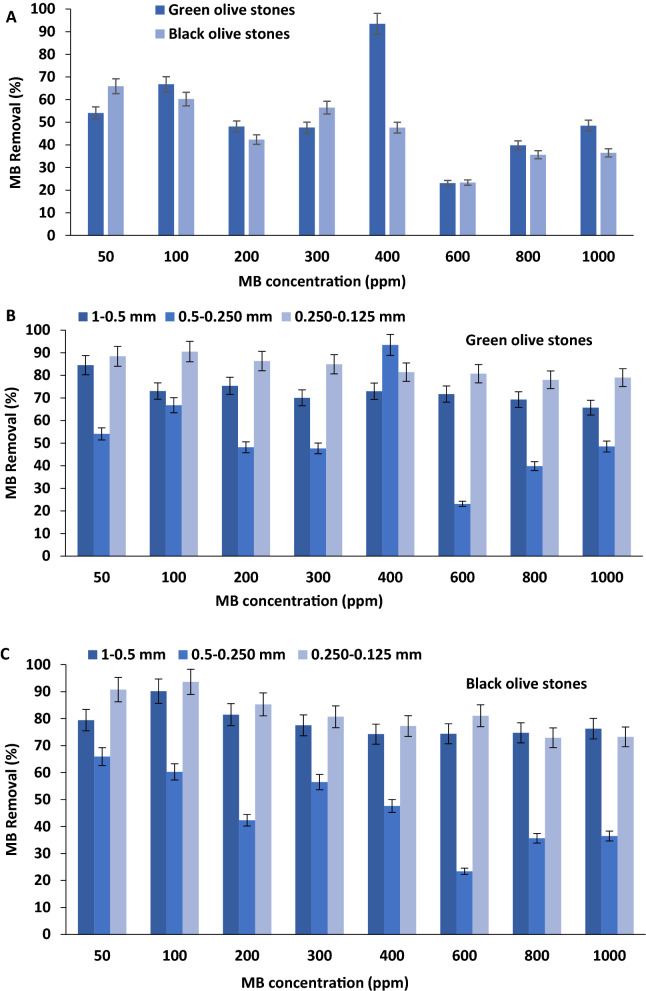
(**A**) Effect of initial dye concentration on the green and black olive stones (Particle size of 0.5–0.250 mm), (**B**) effect of the green olive stones particle size (1–0.5, 0.5–0.250, and 0.250–0.125 mm) (**C**) effect of the black olive stones particle size. Experimental conditions: adsorbent mass: 0.05 g, volume of 50 mL, pH 10, temperature: 25 °C, and contact time: 24 h.

The initial dye concentration affects dye removal efficiency indirectly by either increasing or decreasing the availability of binding sites on the adsorbent. There is an immediate relationship between the percentage removal of dyes and the initial dye concentration in adsorption systems^51^. Generally, an increase in the initial dye concentration in the solution will cause the adsorption sites on the adsorbent surface to become saturated, which eventually leads to a decrease in the removal efficiency. Despite this generalized trend, in many cases, an increase in dye concentration causes the loading capacity of the adsorbent to increase^33^. The results of the percentage removal of MB by both types of olive stones are presented in Fig. 4A–C. From the general trends presented in Fig. 4A, it is evident that the adsorption of MB onto the green olive stones is quite different from the adsorption of the dye onto the black olive stones. The green olive stones show the highest percentage removal of MB at an initial dye concentration of 400 ppm (93.5% removal), while the highest percentage removal of MB for the black olive stones (65.9% removal) is observed at the lowest initial MB concentration (50 ppm). As a result, it can be assumed that the green olive stones are more efficient than the black olive stones in remediating higher concentrated MB wastewater. On the other hand, the black olive stones seem to be a better choice than green olive stones as efficient adsorbents for MB at lower dye concentrations. Furthermore, the lowest adsorption percentages of MB onto the green and black olive stones occurred at initial dye concentrations of 600 ppm. It is shown in Fig. 4A that when the initial dye concentration was 600 ppm, only 23.1% and 23.4% of MB were removed by the green and black olive stones, respectively. These low adsorption percentages are supported by the SEM images discussed above, where the surface morphology of the olive stones did not change significantly from the original state at an initial dye concentration of 600 ppm.

Moreover, the percentage removal of MB onto green olive stones at 50 ppm was 54.1%, then it increased to 66.8% at 100 ppm, followed by decreases to reach 48.1% and 47.6% at 200 ppm and 300 ppm, respectively. After increasing the initial concentration of MB from 300 to 400 ppm, the highest percentage removal was observed and noted at MB initial concentration of 400 ppm. The lowest percentage removal of MB by the green olive stones at 600 ppm was followed by an increase to 39.8% at 800 ppm and another increase to 48.5% at 1000 ppm. These occurrences could be attributed to several factors discussed by Albroomi et al.^52^, as follows: (1) at low initial dye concentrations, the availability of vacant pores, and binding sites on green olive stones are high. However, the fractional adsorption and mass transfer of MB becomes low; leading to the lower percentage removals of MB at initial dye concentrations below 400 ppm, (2) as the initial MB concentrations increase from 300 to 400 ppm, the mass transfer force of MB also increases, leading to high adsorption percentage onto the plenty available binding sites of green olive stones, and (3) as the initial MB concentrations further increases above 400 ppm and particularly at 600 ppm, the ratio of the dye molecules to the available binding sites is at levels that do not support mass transfer. Additionally, at initial MB concentrations above 600 ppm, the mass transfer of MB molecules is higher due to the increased MB to binding sites ratio, however, the number of available binding sites on the green olive stones will decrease and disappear as the MB molecules occupy them. This results in general, lower removal percentages of MB at high initial concentrations.

Similar results were obtained by Vijayakumar and others^53^, who studied the adsorption of Rhodamine-B dye onto perlite. It was found and concluded that the optimization of adsorption percentage can be achieved by increasing the initial dye concentration. Moreover, Terangpi and Chakraborty^51^ examined the effect of initial acid azo dye concentration on their adsorption onto aniline formaldehyde condensate. The two acid azo dyes studied were known as Acid orange 8 (AO8) and Acid violet 7 (AV7). The adsorption of both dyes favored increasing initial concentrations. However, the loading of AO8 took less time and achieved a higher adsorption capacity than AV7 at the same initial dye concentrations.

On the other hand, the black olive stones showed less fluctuation in the percentage removals of MB than green olive stones. The overall trend of the percentage removal of MB by the black olive stones is observed to have less increasing/decreasing fluctuations with each initial concentration than the green olive stones. This can be due to the adsorption process’s heterogeneity and the occurrence of many types of chemical bonding and coordination of MB molecules onto green olive stones as a result of their unique adsorption related functional groups^34^. Furthermore, the highest percentage removal of MB onto the black olive stones was at 50 ppm, followed by decreases at 100 ppm (60.2% removal) and 200 ppm (42.3% removal) only to increase to 56.5% at 300 ppm and decrease again at 400 ppm to reach 47.6%. After the significant drop in the removal of MB by the black olive stones at 600 ppm, it increased at 800 ppm to 35.6% and only slightly at 1000 ppm to 36.5%. The reason behind the high adsorption percentage of MB onto the black olive stones at the lowest initial dye concentration (50 ppm) could be due to the less availability of binding sites on the black olive stones when compared to the green olive stones. This observation was discussed earlier through the SEM images taken for the two types of olive stones. When the pores of the adsorbent material are low in quantity or do not have an efficient surface area for adsorbing pollutants, adsorption will be favored at lower initial dye concentrations. This occurs normally with many kinds of adsorbents, where the mass transfer forces are low due to low initial concentrations of adsorbate molecules. This was demonstrated by Borhade and Kale^38^, who investigated the adsorption of Rhodamine B, Eriochrome black T, and Murexide dyes onto the eggshell powder. The study revealed that at a fixed adsorbent amount and different initial dye concentrations, the adsorption capacity of the eggshell powder is highest at the lowest concentrations for all studied dyes. In another study done by Hameed and El-Khaiary^54^, involving the adsorption of MB onto broad bean peel, it was found that the adsorption of the dye decreased as its initial concentration increased. Opposing these findings, a study done by Zehra et al.^55^, on the removal of Crystal Violet dye using yeast-treated peat found that the adsorption of the dye increased with increasing initial concentration of the dye. Therefore, it can be concluded that the adsorption of MB onto the green and black olive stones is highly dependent on many factors, of which pH, initial dye concentration and adsorbent characteristics are of major importance^33^.

#### Effect of olive stones particle size on the adsorption of MB from water

The adsorbent particle size greatly affects its capacity towards adsorbing pollutants^34^. Increasing the adsorbent’s particle size usually results in a decrease in its adsorptive properties due to a decreased surface area. On the other hand, decreasing the adsorbent’s particle size will result in an enhancement of its adsorptive properties due to an increased surface area^56,57^. However, this is not always the case, as greatly reducing the particle size can result in conditions that are less efficient in terms of sustainability and environmentally friendly remediation techniques. Some of the drawbacks of extremely reducing particle sizes can be a reduction in adsorbent yield and rigidity, as well as an increase in adsorption process costs. Therefore, optimizing the adsorbent particle size is necessary to optimize the conditions for each unique remediation case^58^. For these reasons, MB batch adsorption experiments were conducted using three particle sizes of the green and the black olive stones (1.00–0.50, 0.50–0.250, and 0.250–0.125 mm). In previous experiments, the middle range olive stone size was studied (0.50–0.250 mm) and discussed in detail. Figure 4B,C displays the percentage removals of MB by both adsorbents at different particle sizes (1.00–0.50, 0.50–0.250, 250–0.125 mm).

From the general trends shown in Fig. 4B, it appears that the trends for the percentage removal of MB onto the green olive stones of 1.00–0.50 mm and 0.250–0.125 mm size are similar. However, the trend of the percentage removal of the middle range size (0.50–0.250 mm) is distinguished. The removal of MB onto the adsorbent having the highest and lowest particle sizes fluctuates with low initial MB concentrations (50–400 ppm) until it reaches a relatively constant state. Figure 4B reveals that the adsorption of MB onto the largest particle size of green olive stones is considerably efficient. This is shown from the overall high percentage removals obtained at all the studied initial MB concentrations (percentage removals higher than 65%). Moreover, it can be observed from Fig. 4B that the highest and lowest percentage removals of MB onto the green olive stones of 1.00–0.50 mm size were obtained at initial dye concentrations of 50 ppm and 1000 ppm, respectively. The green olive stones adsorbed 84.5% MB at 50 ppm MB and 65.7% MB at 1000 ppm (*P* value ≥ 0.05). In comparison to the largest adsorbent’s particle size studied (1.00–0.50 mm), the adsorption of MB onto the smallest particle-sized adsorbents studied (0.250–0.125 mm) fluctuates less with a change in dye concentrations. Figure 4B also shows that the green olive stones of the smallest particle size achieved the highest and lowest percentage removals of MB at initial MB concentrations of 100 ppm and 800 ppm, respectively (*P* value < 0.05). It can be concluded from Fig. 4B that the adsorption of MB onto the green olive stones favors decreasing particle size due to the increase in surface area for binding.

On the other hand, Fig. 4C shows the percentage removals of MB by the black olive stones in different particle sizes. Similar to Fig. 4A, the general trends of the black olive stones at the largest and smallest particle sizes appear to be similar. It appears that the black olive stones achieved the highest and lowest percentage removals of MB at initial dye concentrations of 100 ppm and 400 ppm, respectively. An MB percentage removal of 90.2% was obtained at 100 ppm and 74.2% at 400 ppm. In comparison to the overall percentage removals of MB by green olive stones (Fig. 4B) at the same particle size (1.00–0.50 mm), it can be concluded that the black olive stones are more efficient in remediating MB than green olive stones at the studied particle size (*P* value ≥ 0.05). This is due to the overall higher remediation of MB by the black olive stones of size 1.00–0.50 mm (percentage removals higher than 74%). Moreover, the presentation of the percentage removal of MB onto the black olive stones of the size range of 0.250–0.125 mm is shown in Fig. 4C. The black olive stones obtained the highest and lowest percentage removals of MB at initial MB concentrations of 100 ppm and 800 ppm, respectively (*P* value < 0.05). However, the black olive stones accounted for slightly higher percentage removal of MB than green olive stones (Fig. 4B) at 100 ppm (93.6% for the black and 90.5% for the green olive stones). On the contrary, at an initial MB concentration of 800 ppm, green olive stones (Fig. 4B) still achieved higher percentage removal (78%) of the dye than the black olive stones (72.9%). Furthermore, the green olive stones obtained higher percentage removals of MB at 200, 300, 400, 800, and 1000 ppm than the black olive stones at the smallest particle size (Fig. 4B,C). This can lead to the conclusion that the green olive stones could be more efficient than the black olive stones in remediating a wider range of MB concentrations at a decreasing particle size.

Figure 4B,C shows that the green olive stones adsorbed higher percentages of MB at 100, 200, 400, 800, 1000 ppm than the black olive stones in the middle range particle size. Therefore, as with the results obtained for the adsorbent particle size of 0.250–0.125 mm, green olive stones are more effective than the black olive stones (size range of 0.50–0.250 mm) in decolorizing a wider range of MB concentrations. Overall, the results obtained for the adsorption of MB onto the green and black olive stones at three particle sizes reveal that the adsorption increases with decreasing particle size of the green olive stones in a wide range of initial dye concentrations. In contrast, the black olive stones exhibited higher adsorption of MB than green olive stones at increasing adsorbent particle size.

Similar results were obtained by Wong et al.^59^, studying the effect of particle size on the adsorption of five acidic dyes onto chitosan. For all the studied dyes, the adsorption capacity of chitosan increased with decreasing particle size, which indicated the importance of the surface area for adsorption. In addition, the adsorption of albumin-bonded bilirubin onto granular activated carbon of different micrometer sizes was shown to favor decreasing particle size due to increased surface area and pore volumes^60^. Maghri et al.^61^ studied the adsorption of MB onto corn stalks and Mytilus Edulis shells at different particle sizes. The adsorption of MB was enhanced with decreased particle sizes for both adsorbents. However, both adsorbents at the study obtained the highest MB removals at different particle sizes. Corn stalks adsorbed more dye at a size range of 0.08–0.2 mm while Mytilus Edulis shells adsorbed more MB at a size range of 0.056 mm and smaller. These results indicate the uniqueness of adsorbents toward a pollutant due to the various factors such as surface morphology and chemistry, which play a major role in adsorption processes.

#### Effect of temperature on the adsorption of MB onto olive stones and thermodynamics

**Figure 5:**
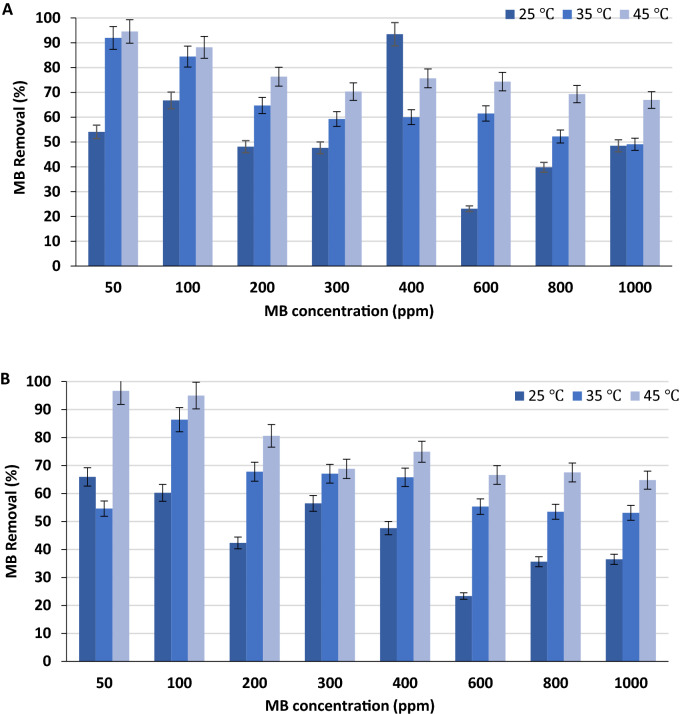
Effect of temperatures (25, 35, and 45 °C) on the adsorption of MB onto (**A**) green olive stones and (**B**) black olive stones. Experimental conditions: adsorbent mass: 0.05 g, particle size 0.50–0.25 mm, volume: 50 mL, pH 10, and contact time: 24 h.

Adsorption processes are generally exothermic; however, some of them are endothermic. This means that the adsorption capacity of the adsorbent may increase or decrease with increasing temperature. An increase in the adsorption capacity with temperature indicates an endothermic adsorption process, while a decrease in the adsorption capacity with increasing temperature indicates an exothermic process. Temperature plays an important role in controlling the strength of the adsorptive forces between the adsorbent and adsorbate molecules^32,33^. The effect of temperature on the remediation of MB onto the green and black olive stones was studied under 25, 35, and 45 °C. The percentage removals of MB by the adsorbents at the study are presented in Fig. 5A,B. As shown in Fig. 5, both olive stones achieved higher removal percentages of MB when the temperature was increased from 25 to 45 °C. The highest adsorption of MB onto both types of olive stones (Fig. 5) occurred in 45 °C (*P* value < 0.05). This indicates that the adsorption process is endothermic and favors high temperatures^52^. More specifically, the trend observed with the adsorption of MB onto the green olive stones at 35 and 45 °C (Fig. 5A) shows the increasing adsorption with temperature, where adsorption was the highest at lower initial MB concentrations. Comparing the trends for the adsorption of MB onto the black olive stones at 35 and 45 °C (Fig. 5B), it is evident that the adsorption trends are slightly different. It is shown that the percentage removal of an initial concentration of 50 ppm MB onto the black olive stones at 35 °C was much lower than the percentage removal of an initial concentration of 50 ppm MB at 45 °C. A recent study obtained similar results where the adsorption of Congo Red (CR) dye onto Ca-bentonite was investigated at temperatures varying from 20 °C to 50 °C. The investigators found that the removal percentage of CR was the highest at 50 °C^62^. The adsorption of Rhodamine B onto perlite was investigated by three different temperatures of 30, 40, and 50 °C. From the results of the study, Rhodamine B showed facilitated adsorption onto the adsorbent when the temperature increased from 30 to 50 °C^53^. Al-Degs et al.^63^ also studied the effect of temperature on the adsorption of C.I. Reactive Blue 2 and C.I. Reactive Yellow 2 dyes onto activated carbon. The study revealed that the adsorption capacities of the dyes favored higher temperatures. The study suggested that the adsorption process is endothermic where the dye molecules need energy in order to move around and penetrate deeper into the micro-pores of activated carbon at increasing temperatures. The MB at the study shows similar behavior towards the olive stones, where energy from the increasing temperature would enhance the adsorption process.

### Adsorption isotherms models

The adsorption isotherms describe the relationship at the equilibrium of the amount of the adsorbed material onto the adsorbent (q_e_) and the equilibrium concentration in the bulk fluid (C_e_) at constant temperature and pH. Therefore, the critical optimization of the pathways of adsorption mechanisms, capacities, and surface properties of adsorbents, as well as adsorption systems can be achieved^32,64^. For these reasons, four isotherm models are known as Langmuir, Freundlich, Dubinin–Radushkevish, and Temkin were tested to determine the applicability of the green and black olive stones in remediating MB from water. The calculated isotherm constants and equation parameters related to the adsorption MB onto the olive stones are presented in Table 2. Upon general observation, all the isotherm models for the two adsorbents show high R^2^ values, which means that the adsorption of MB onto the green and black olive stones could follow all models^65^. However, the green olive stones obtained the highest R^2^ value with the Temkin isotherm model, whereas the black olive stones showed the highest R^2^ value with the Langmuir isotherm model.

**Table 2:** Equation parameters of Langmuir, Freundlich, Dubinin–Radushkevish, and Temkin isotherm models for the adsorption of MB onto two adsorbents with a variety of initial MB concentrations and temperatures at 25, 35 and 45 °C.

Adsorbent	Langmuir model $\frac{{C_{e } }}{{q_{e} }} = \frac{1}{{K_{L} \cdot Q_{0} }} + \frac{{C_{e} }}{{Q_{0} }}$			Freundlich model $Logq_{e} = LogK_{f} + \frac{1}{n}LogC_{e}$			
	Q_0_ (mg/g)	K_L_ (L/mg)	R^2^	*n*	1/*n*	K_f_ ((mg/g) (L/mg)^1/n^)	R^2^
**25** **°C**							
Green olive stones	588.2	799.7	0.8	1.5	0.7	4.4	0.8
Black olive stones	476.2	858.0	1.0	1.7	0.6	6.8	0.9
**35** **°C**							
Green olive stones	626.0	2164.9	0.8	1.5	0.5	20.8	1.0
Black olive stones	666.7	2180.1	0.9	1.5	0.7	7.7	0.8
**45** **°C**							
Green olive stones	909.1	4959.6	0.9	1.8	0.6	22.7	1.0
Black olive stones	909.1	4959.6	0.7	1.8	0.6	22.7	1.0

	Dubinin–Radushkevich model $q_{e} = q_{D} \cdot \exp \left( { - B_{D} \left[ {R \cdot T \cdot \ln \left( {1 + \frac{1}{{C_{e} }}} \right)} \right]^{2} } \right)$			Temkin model $q_{e} = \frac{RT}{{b_{T} }}lnA_{T} + \left( {\frac{RT}{{b_{T} }}} \right)lnC_{e}$			
	B_D_ (mol^2^/KJ^2^)	q_D_ (mg/g)	R^2^	B (J/mol)	b_T_	A_T_ (L/mg)	R^2^
**25** **°C**							
Green olive stones	0.0	201.1	0.9	− 94.8	− 26.1	2.7	1.0
Black olive stones	0.0	129.1	0.7	58.6	42.3	0.1	0.9
**35** **°C**							
Green olive stones	0.0	388.0	0.8	191.7	13.4	2.7	1.0
Black olive stones	0.0	318.5	0.9	126.4	191.7	14.2	0.9
**45** **°C**							
Green olive stones	0.0	499.9	0.8	− 455.4	− 5.8	2.7	0.8
Black olive stones	0.0	499.9	0.8	176.9	15.0	0.1	0.8

R is the universal gas constant (8.314 J/mol K), T is the temperature, b_T_ is the Temkin isotherm constant, A_T_ is the Temkin isotherm equilibrium binding constant, $q_{D}$ is the Dubinin–Radushkevich adsorption capacity in (mg/g), *B*_*D*_ is the free energy coefficient of the adsorption.

The R^2^ values of the Langmuir isotherm model for the green and black olive stones (Table 2) indicate that both adsorbents follow the model’s theory. This means that MB molecules form a homogeneous monolayer with no interactions of molecules with adjacent sites^39^. The monolayer adsorption capacity (Q_0_) of the green olive stones was found to be slightly higher than the black olive stones with values of 588.2 mg/g and 476.2 mg/g, respectively. Accordingly, from the values of the Langmuir isotherm constant (K_L_) it can be concluded that the adsorption of MB onto the black olive stones is more favorable than green olive stones^66^. Table 2 also shows that the adsorption of MB onto the green and black olive stones follow the Freundlich isotherm model to a great extent. The high R^2^ helps in drawing the conclusion that the adsorption of MB on the studied adsorbents is reversible and non-ideal with the formation of non-uniform multilayers of adsorbed materials on the surface of the adsorbent^53^. The values of *n* related to the Freundlich isotherm model for the green and black olive stones are 1.5 and 1.7. The value of *n* is of great importance when an indication of the curvature of the isotherm is needed. If an *n* value of 1 is obtained, then the adsorption is linear, if *n* is bigger than 1 then the adsorption is a physical process and favorable and if the value of *n* is smaller than 1, the adsorption is a chemical process and unfavorable^67,68^. According to the results of the study, both the adsorption processes of MB (onto the green and black olive stones) follow a favorable physical process. The other Freundlich constant derived from *n* is 1/*n*. According to Terangpi and Chakraborty^51^, a value of 1/*n* greater than 1 indicates a cooperative adsorption process while a value of 1/*n* less than 1 indicates a less cooperative adsorption process. Moreover, as the value of 1/*n* decreases, the adsorption becomes more heterogeneous and that at low adsorbate concentrations, the adsorption is more efficient^38,68^. The values of 1/*n* for the green and olive stones are very close to each other and are both less than 1 (0.7 for the green olive stones and 0.6 for the black olive stones), which indicates that the adsorption of MB onto olive stones is not very cooperative, heterogeneous and efficient at low MB concentrations. The adsorption capacity for MB onto the olive stones is represented by the Freundlich constant K_f_^64^. The results obtained show that the black olive stones have a higher adsorption capacity (6.8 mg/g) for MB than the green olive stones (4.4 mg/g) when referring to the Freundlich isotherm model. From Dubinin–Radushkevich energy constant (B_D_) values obtained for the green and black olive stones in Table 2, it appears that the adsorption of MB is an energy-free process. Furthermore, the adsorption capacity (q_D_) values of the Dubinin–Radushkevich isotherm model (Table 2) for the adsorbents in this study show that the green olive stones can obtain higher adsorption of MB than the black olive stones. From the R^2^ values of the Dubinin–Radushkevich model, the green olive stones give a better description of the model than the black olive stones for the adsorption of MB. The Temkin adsorption isotherm model’s R^2^ values indicate that the model gives great fitting to both types of adsorbents with better fitting for the green olive stones. This means that the adsorption process of MB depends on the heat of adsorption. In addition, the heat of sorption constant (B) value for green olive stones is negative, which means that the adsorption of MB onto green olive stones is a physical endothermic process. On the other hand, the black olive stones obtained a high value for the Temkin’s heat of sorption constant and provide a chemical exothermic adsorption process of MB^32,38,65^.

The applicability of the adsorption of MB onto the green and black olive stones to Langmuir, Freundlich, Dubinin–Radushkevich, and Temkin isotherm models with varying temperatures (35 °C and 45 °C) was studied. As with the previous experiment, the isotherm constants for the green and black olive stones involved in the adsorption of MB at different temperatures (35 and 45 °C) were calculated. The linearity and R^2^ values of the isotherm models is clear in Table 2. From Table 2, all the isotherm models for the two adsorbents show relatively high R^2^ values at all temperatures, which means that the adsorption of MB onto the green and black olive stones could follow all models^39^. However, the green olive stones exhibit the highest R^2^ value with the Langmuir isotherm model at 35 °C, while with the Freundlich isotherm model the highest R^2^ for green olive stones is at 45 °C. In addition, Dubinin–Radushkevich and Temkin isotherm model for green olive stones show the highest R^2^ for green olive stones at 25 °C. The black olive stones have the highest R^2^ value at 25 °C with the Langmuir isotherm model and at 45 °C with the Freundlich isotherm model. The highest R^2^ for the black olive stones with Dubinin–Radushkevich and Temkin were observed at 35 °C. These results indicate that varying the temperature results in changes in the mechanism of the adsorption of MB onto both types of olive stones^65^.

The R^2^ value of the green olive stones increased from 0.8 to 0.9 when the temperature of the adsorption increased from 35 to 45 °C. However, the R^2^ value for the black olive stones decreased from 0.9 to 0.7 when the temperature of the adsorption increased from 35 to 45 °C. This could indicate that the adsorption of MB onto the green olive stones follows the Langmuir isotherm model better at higher temperatures while the adsorption onto the black olive stones best fits the model at lower temperatures^38^. The monolayer adsorption capacity (Q_0_) of both adsorbents towards MB increased when the temperature was increased from 35 to 45 °C. However, the more increase in Q_0_ was observed for the green olive stones than the black olive stones. This increase in the monolayer adsorption capacity of the adsorbents resulted in the increase of Langmuir’s adsorption favorability constant (K_L_) for the adsorbents with increasing temperature^39,66^. Table 2 shows the isotherm constants related to the Freundlich isotherm model. The high R^2^ values for the green and black olive stones at both temperatures (35–45 °C) show the fitting of the adsorption mechanism of MB to the Freundlich isotherm model. This means that MB could form reversible uniform multilayers on the surface of the adsorbents^67^. According to the values of *n* obtained for the Freundlich isotherm model at 35 °C and 45 °C, the adsorption of MB onto the green and black olive stones is a favorable physical process at both temperatures. The value of *n* at 45 °C (1.8) is higher than the value at 35 °C (1.5), which indicates the increased favorability of the adsorption at 45 °C^68^. The value of 1/*n* related to the Freundlich isotherm model for the green olive stones increased slightly from 0.5 to 0.6 with the increase in temperature. On the other hand, the value of 1/*n* for the black olive stones decreased slightly from 0.7 to 0.6 with an increase in temperature. Both adsorbents at study showed values of 1/*n* less than 1 which means that the adsorption process is not very cooperative. The decrease in 1/*n* for the black olive stones with increasing the temperature means that the adsorption of MB becomes more heterogeneous and less cooperative as the temperature increases. The adsorption of MB onto the black olive stones is more efficient at low adsorbate concentrations^69^. This is a confirmation of the results obtained from Fig. 4B, where the highest percentage removals of MB onto the black olive stones at all temperatures were obtained at lower MB initial concentrations. The Freundlich constant (K_f_) value for the green olive stones did not significantly change when the temperature increased from 35 to 45 °C. Indicating very similar adsorption capacity towards MB at both studied temperatures. However, the K_f_ value of the black olive stones increased from 7.7 to 22.7 mg/g when the temperature increased from 35 to 45 °C. This indicates the significant effect of temperature on the adsorption capacity of the black olive stones^70^. The Dubinin–Radushkevish isotherm model constants are presented in Table 2. The R^2^ values show the applicability of the adsorption process of MB onto the green and olive stones. The energy constant (B_D_) values for the Dubinin–Radushkevich isotherm model indicated that the adsorption process for both adsorbents was an energy-free process. The Dubinin–Radushkevich adsorption capacity constant (q_D_) demonstrates that at 35 °C, the green olive stones exhibited a higher capacity (388 mg/g) than the black olive stones (318.5 mg/g) for the removal of MB. Although at 45 °C, both adsorbents show equal capacities to adsorb MB (499.9 mg/g). The R^2^ values for the Temkin isotherm model reveal that the adsorption of MB onto the green olive stones could also be described by the model. Moreover, the value of the heat of sorption (B) for the green olive stones at 35 °C is positive while it is negative at 45 °C. As mentioned previously (Table 2), the B_D_ value for the adsorption of MB onto the green olive stones at 25 °C was negative. This means that the adsorption of MB onto the green olive stones according to the Temkin’s mechanism of adsorption could be physical and endothermic at the lowest and highest studied temperatures, while chemical and exothermic at 35 °C. Comparing the black olive stones to the green olive stones, they exhibited high positive values of B_D_ at all studied temperatures, indicating the chemical and the exothermic adsorption process of MB^62,65^.

In addition, the MB adsorption capacities of the green and the black olive stones were compared with other previous studies; it is concluded that their adsorption capacities were much higher than other adsorbents^71–79^.

### Adsorption thermodynamics

In adsorption systems, thermodynamic concepts such as enthalpy, entropy, and Gibbs free energy can be calculated along with the adsorption isotherms to further characterize the behavior of reactions and provide insights towards the tendency of proposed clean-up measures of many environmental pollutants by adsorption technique^35,36^. Calculation of the thermodynamics was carried out for the adsorbents at study at 25, 35, and 45 °C where MB concentration varied as discussed previously Eqs. (3) and (4). The ∆G° values for both adsorbents were quite similar which were − 16.6 kJ/mol, − 19.6 kJ/mol, and − 22.6 kJ/mol at 25 °C, 35 °C, and 45 °C, respectively. While the values of ∆H° and ∆S° were 72 kJ/mol and 297.2 J/K mol^−1^, respectively. According to the negative values obtained for Gibbs free energy, it can be concluded that the adsorption of MB onto the green and black olive stones was spontaneous at all the studied temperatures. Furthermore, the positive values for the entropy confirm the spontaneity and high level of disorder of the adsorption process. The positive values obtained for the enthalpy indicate that the adsorption process of MB onto both olive stones is endothermic. This was verified previously from the results of the effect of temperature on the adsorption of MB onto both types of olive stones.

To assess the significance of the results obtained in this study, analysis of variance (ANOVA) was conducted for all experiments (effect of pH, temperature, and adsorbent particle size on the adsorption of MB onto olive stones). The single-factor ANOVA test was used for the effect of pH and particle size on the adsorption of MB onto olive stones because the temperature and MB concentration was constant. In contrast, a two-factor ANOVA test was conducted to address the relationship between the initial MB concentration and temperature. The results of the single factor ANOVA test for the impact of pH and adsorbent particle size on the removal of MB showed a *P* value of ≥ 0.05 for both types of olive stones. Therefore, the results are not significantly different. However, the two-factor ANOVA results of the effect of concentration and temperature on the removal of MB onto the green olive stones show a significant difference at a p-value of 0.01. Furthermore, the black olive stones showed a significant difference in the effect of concentration and temperature on MB adsorption with a p-value of 0.000033.

### Application of the black and green olive stones to a real wastewater sample

A textile laundry wastewater was used as an adsorption medium for investigating the effect of the black and green olive stones in MB removal from real wastewater. The characteristics of the collected wastewater used in this study were pH (7.3), conductivity (711 μS/cm), chemical oxygen demand (COD) (143 mgO_2_/L), biological oxygen demand (BOD_5_) (88.3 mgO_2_/L), total organic carbon (TOC) (81.4 mg/L), the phosphate content (153.1 mg/L), absorbance at 663 nm (0.049) and total suspended solids (TSS) (222 mg/L). The wastewater sample was then subjected to MB contamination with a concentration of 600 mg/L. The adsorption capacities (q_e_) for MB were 458 and 525 mg/g for the black and green olive stones, respectively. These are corresponding to 76.33% and 81.45% removal percentages, respectively. This indicates the great potential application of the black and green olive stones in removing MB from dye wastewater.

### Desorption studies

It is very important to study the desorption process and mechanisms of the adsorbed MB on the black and green olive stones. Reusing the spent adsorbents are considered as an important economic aspect in reducing material costs. As a sustainable development approach, the desorption study was limited only to using ethanol and acetic acid in order not to produce hazardous leachates^74^. It was revealed that the desorption removal capacity increased with the increase in acetic acid concentration to 10%vol. The total desorption removal capacities of the MB-loaded black and green olive stones were found to be 92.5 and 88.1%, respectively. In view of that, it was proposed that the forces of attraction between MB and the black and green olive stones were weak, and they were almost the same strength as physisorption. However, the desorption removal capacity was higher for the black olive stones.

Mouni et al., 2018^74^ studied the desorption studies with water. It was indicated that the adsorbent successfully retain MB, even after several cycles. After four cycles, the MB adsorption capacity decreased from 56 to 23%. Pathania et al.^79^ performed the desorption studies with 1% HCl, H_2_SO_4_ and NaOH. Different desorption media were studied. 100 mg of the activated carbon developed from *Ficus carica bast* was saturated with 5 mg/L (50 mL) of MB. 84% of MB were desorbed using HCl as a desorption medium. However, the adsorption efficiency was reduced to 45% after six cycles.

## Conclusion

Establishing innovative, cost-effective, and environmentally friendly wastewater treatment technologies is vital. This is due to the well-recognized increase in global population and development, which pose extreme pressure on textile industries to meet the human demands for textiles. For these reasons, utilizing olive stones as decoloring agents for textile wastewater is very beneficial because olive stones are considered as agricultural waste. To optimize the adsorption process, a detailed study of the adsorbent’s nature was done by SEM, FTIR, CHN elemental analysis, pH_solution_, as well as by determining bulk and particle densities. The effect of operating conditions in terms of pH, temperature, particle size, and initial concentration was studied in order to determine their impact on the removal of MB using olive stones. Both adsorbents (black and green olive stones) achieved the highest percentage removals at a pH value of 10 and a temperature of 45 °C. However, the green olive stones were found to be more efficient in remediating high MB concentrations from water than the black olive stones. A removal percentage of 93.5% occurred for the green olive stones at an initial MB concentration of 400 ppm, compared to a removal percentage of 47.5% for the black olive stones at the same MB concentration. The adsorption isotherm models such as Langmuir, Freundlich, Dubinin–Radushkevich, and Temkin were studied which helped to obtain information about the adsorption mechanisms. By studying adsorption thermodynamics, the adsorption of MB onto olive stones was found to be endothermic, spontaneous, and favorable. The results of the study showed that the agricultural waste of olive stones could be harnessed and used without modifications or technical works to serve the important purpose of environmental cleanup. Due to the excellent findings of the study, the adsorbents could be used at large scale textile wastewater treatment plants, where they would ensure less harmful impacts on the environment and sustainability.
